# Digital empathy in behaviour change interventions: A survey study on health coach responses to patient cues

**DOI:** 10.1177/20552076231225889

**Published:** 2024-03-15

**Authors:** E Rey Velasco, Z Demjén, TC Skinner, on behalf of the Impact Diabetes B2B Collaboration Group

**Affiliations:** 1 Liva Healthcare, 86985 Københavns Universitet Institut for Psykologi, Copenhagen, Denmark; 2 Department of Psychology, 4321 University of Copenhagen, Kobenhavn, Denmark; 3 UCL Centre for Applied Linguistics, 4919 University College London, London, UK

**Keywords:** Health communications, telehealth, psychology, digital health, behaviour change, lifestyle

## Abstract

**Introduction:**

Digital health coaching interventions for behaviour change (BC) are effective in addressing various health conditions. Implementing these requires accurate descriptions of components and health coaches (HC) delivery methods, alongside understanding patients’ perceptions of these interactions. The HC–patient relationship significantly influences BC outcomes. Here, empathy is an important driver that enables HCs to offer tailored advice that resonates with patients’ needs, fostering motivation. Yet, defining and measuring empathy remains a challenge. In this study, we draw on various BC frameworks and Pounds’ empathy appraisal approach to categorise HCs responses to patient cues and explore the interplay between empathy and BC.

**Methods:**

Using a two-round survey, we collected responses from 11 HCs to 10 patient messages from the Bump2Baby and Me trial in a simulated interaction. We analysed 88 messages to identify empathic responses and behaviour change techniques.

**Results:**

Patients’ implicit empathy opportunities showed higher response rates than explicit ones. HCs prioritised positive reinforcement and employed various strategies to achieve similar objectives. The most common empathic response was ‘Acceptance’ for patients’ implicit positive expressions of self-judgement. HCs emphasised relatedness-support and competence-promoting techniques for implicit negative feelings and judgements, such as ‘Show unconditional regard’ and ‘Review behaviour goals’, and ‘Action planning and Problem-solving’ techniques to address explicit negative appreciations and feelings.

**Conclusion:**

The use of different techniques with the same objective highlights the complexity of BC interactions. Further research is needed to explore the impact of this variability on patient outcomes and programme fidelity.

## Introduction

In healthcare, translating research evidence into practical applications remains a critical challenge, particularly when implementing evidence-based guidelines and interventions.^1^ The fidelity assessment of these interventions, especially in behaviour change (BC) programmes, faces challenges due to the heterogeneity in methodological approaches.^2^ BC interventions play a pivotal role in addressing diverse health issues, including chronic diseases, mental health disorders, and lifestyle modifications,^3,4^ and their successful implementation on a large scale has profound implications for public health.^5^ An accurate description of BC programmes serves to identify crucial intervention components and expand theory and ensures their applicability across various healthcare settings and populations.^6^ This article seeks to advance the study of these crucial components by offering a fresh perspective focused on empathy in a digital context, given the growing use of digital technologies to deliver BC programmes.

By elucidating the successful implementation of BC interventions on a large scale and its profound implications for public health, the article seeks to provide a clear understanding of the crucial components and theoretical underpinnings. Addressing the heterogeneity in methodological approaches, we explore how accurate descriptions of BC programmes can serve to identify key intervention components, expand theory, and ensure the applicability of these programmes across various healthcare settings and populations. This comprehensive exploration aims to enhance the readability of the subsequent sections and offer a strong frame of reference for the reader.

The Behaviour Change Techniques (BCTs) taxonomy, consisting of 93 techniques across 16 domains, was created through BC expert consensus.^7^ It serves as a standardised framework for classifying BCTs, which represent the ‘active ingredients’ of interventions designed to support individuals in modifying health-related behaviours, such as diet and physical activity^8^ or smoking cessation.^9^ Researchers can use the taxonomy as a common language to identify effective BCTs, replicate interventions, learn best practices, and assess intervention fidelity.^10,11^

**Figure 1.**
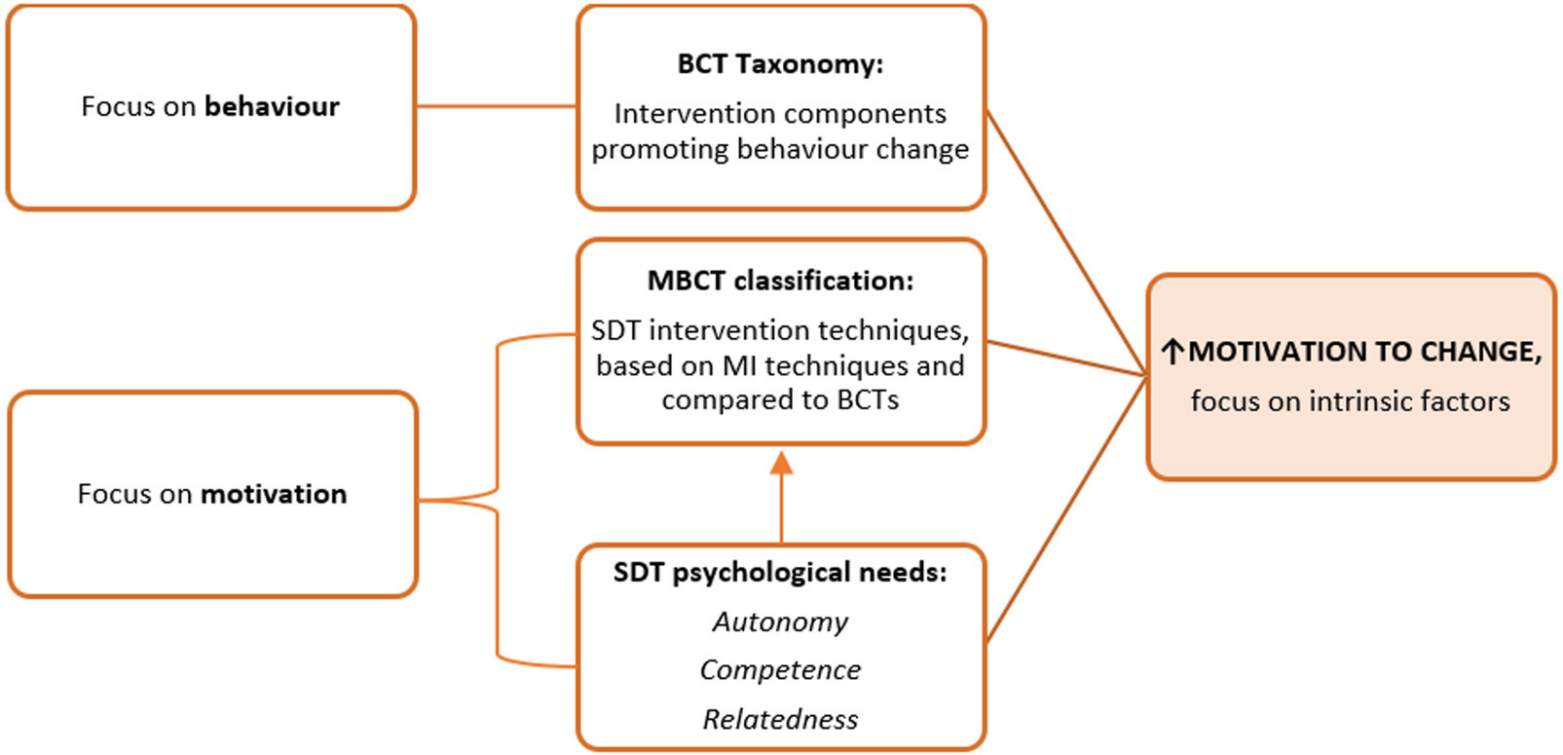
Illustration of the interrelation among the BCT, MI, and SDT frameworks, aimed at optimising motivation for behaviour change in an individual. BCT: behaviour change technique; MBCT: motivational behaviour change technique; MI: motivational interviewing; SDT: self-determination theory.

Following the creation of the BCT taxonomy, additional, and overlapping techniques specific to other theoretical frameworks, such as self-determination theory (SDT)^12^ and motivational interviewing (MI)^13^, have been identified. SDT establishes the psychological factors and processes that underlie motivation in terms of three main psychological needs: autonomy, competence, and relatedness, and their connection to BC. The fulfilment of these three needs ensures an optimal motivation to change.^14^ MI shifts the focus from external consequences, such as rewards or punishments, to the intrinsic consequences of BC to support long-term changes. This approach aligns with SDT's emphasis on identifying personally meaningful reasons for the individual to pursue BC.^15^ As a result, Teixeira et al.^16^ expanded MI by incorporating several BCTs (e.g. open-ended questions, reflective listening, and providing feedback) and the three psychological needs classification from SDT into a 21 motivation and behaviour change techniques (MBCT) taxonomy. Integrating these key elements into BC interventions strengthens autonomous motivation and improves the effectiveness of these programmes (Figure 1).

Health coaching is an effective and scalable practice for supporting BC.^4,17^ Despite the identification of the theoretical frameworks and techniques employed, the replication and implementation of effective coaching interventions faces challenges. Accurate descriptions of how the intervention is delivered, including the strategies, language, and delivery styles, at all intervention stages, as well as how these are perceived by the patient, is essential.^18^ Here, the patient–provider relationship is a decisive factor for BC outcomes.^19^ While the coach's empathy is an important driver in this relationship, it is often overlooked or measured inaccurately because of inconsistencies in its definitions, treatments, and evaluations.^20^ Thus, the role of empathy in BC demands a more explicit description and fidelity check of coach responses for every healthcare intervention.^21^

In clinical practice, empathy is the ability to ‘feel with’ the patient, that is, ‘to understand and participate in another person's feelings and emotional state’.^22^ In this context, the language choices that shape patient-provider communication significantly influence patient outcomes,^23^ prompting researchers to explore health interactions through various lenses. While some rely on quantitative outcomes like clinical measurements or questionnaires, qualitative methods emphasising conversational topics or behaviours are generally preferred.^24^ Pounds^25^ developed an empathy appraisal approach for the expressions of empathy grounded on Systemic Functional Linguistics (SFL), a linguistic theory developed by Halliday^26^ that studies the interplay between language structure, meaning, and context. Furthermore, as described by Martin,^27^ the appraisal theory within the SFL framework provides an additional lens through which linguistic resources are analysed to understand how individuals appraise and express their attitudes and emotions in discourse. Pounds’ framework outlines seven forms of empathy opportunities (EOs), or patient's expressions and cues, that healthcare professional (HCPs) can recognise and respond to empathetically (see Table 1). These EOs can be differentiated as implicit, referring to statements from which an underlying emotion that has not been explicitly expressed might be inferred, or explicit, regarding statements that are plausibly associated with an emotion.^28^

**Table 1.** Description and examples of patient's EOs adapted from Rey Velasco et al.^31^

EO	Category	Example
**EO1**	EXPLICIT EXPRESSIONS OF NEGATIVE FEELINGS, such as an emotive behaviour or a mental state	‘I cried when I found out’
**EO2**	IMPLICIT EXPRESSION OF NEGATIVE FEELINGS through reference to a negative experience, such as fear, confusion, anxiety, or sadness	‘It's been 3 days and I haven’t heard back from my GP’
**EO3**	EXPLICIT EXPRESSION OF NEGATIVE JUDGEMENT (others or self)	‘She is such an irresponsible person’
**EO4**	IMPLICIT EXPRESSION OF NEGATIVE JUDGEMENT (others or self)	‘I could have done better’
**EO5e**	EXPLICIT EXPRESSION OF POSITIVE SELF-JUDGEMENT	‘I am a good father’
**EO5i**	IMPLICIT EXPRESSION OF POSITIVE SELF-JUDGEMENT	‘I am eating healthier than ever!’
**EO6**	EXPLICIT EXPRESSION OF NEGATIVE APPRECIATION (things, events, actions).	‘The dinner was so boring’
**EO7**	IMPLICIT EXPRESSION OF NEGATIVE APPRECIATION (things, events, actions)	‘I am not sure this is something for me’

EO: empathy opportunity.

While research on empathy in healthcare interactions has predominantly focused on live or synchronous settings, such as face-to-face consultations or telemedicine video calls, there is a significant gap in empathy dynamics within asynchronous written interactions.^29^ With the increasing popularity of digital communication platforms in healthcare,^30^ comprehending how empathy is expressed, perceived, and experienced is essential, as it influences patient satisfaction, trust, and outcomes. Furthermore, despite the acknowledged significance of empathy as a catalyst for behaviour change, the existing literature lacks research directly correlating empathy categories with BCTs.

This article aims to explore different providers’ expressions of empathy in asynchronous text-based interactions in response to messages extracted from a digital BC coaching intervention. Drawing on Pounds’ framework, which categorises patient EOs and HCP empathic responses, we coded coach responses to EOs. While there is scarce research on the identification of these EOs in written health interactions,^31,32^ to our knowledge, there is no research work on the connection between provider's responses and EOs. In addition, we identify the techniques in those empathic responses to explore the relationship between empathy and BC from a novel coding perspective. This perspective aims to establish a foundation to measure the fidelity of BC interventions delivery and provider training in an asynchronous setting, contributing to the advancement of digital health coaching practices.

## Methods

### Survey participants

This research is part of a randomised controlled trial study where Liva Healthcare A/S (Liva) is the tele-health coaching services provider. Our recruitment criteria were (1) to be a Liva HC and (2) to provide coaching services in English. We recruited participants via a private Slack®^33^ network channel that Liva HCs use on a daily basis. At the time of recruitment, 35 HC met our criteria. We posted a message with survey details and an email address for HCs to contact if they wished to participate. We recruited 11 HCs, of which 1 dropped out and 1 did not complete the full survey.

### Coaching programme characteristics

Liva HCs are registered dietitians or nutritionists, and have a health-related degree (nursing, physiotherapy, midwifery, and health psychology) and a basic knowledge of the BC framework at the time of enrolment. Subsequently, they receive an evidence-based 13 h of training on BC theories such as SDT and MIT, as well as coaching strategies and BCTs, and programme-specific guidance where necessary (i.e. in the Bump2Baby and Me trial, which is the source of our patient survey messages as explained below, HCs received a total of 27.5 h of training). Among other methods, Liva HCs use Whitmore's Goal-Reality-Options-Wrap-Up model (GROW), an autonomy-promoting tool to guide the patient in developing their own conclusions.^34^

### Survey design

We designed a two-round online survey to examine how participants (HCs) responded to the same 10 patient messages, see details below. Once participants agreed to participate, we sent them a document containing a selection of the techniques (Appendix 1) that were applicable to the intervention and survey settings. After a 2-week period, each participant received a link for the first round, during which they were asked to select those techniques that were more suitable to a text-based response, based on their coaching expertise. Once all the participants had responded, we then identified the techniques chosen by ≥50% of participants and used them to guide their responses in the next round. In the second round, we asked participants to compose a text response, as they would do when acting as a HC, including as many techniques as they deemed necessary. In both rounds, there was an open-response box for comments and suggestions on the inclusion or removal of particular techniques. The recruiting and start of the study were conducted on an ongoing basis, and each participant had 2 weeks to complete each round of the survey. The first participant received the techniques document on 1 September 2022, and the last participant completed the second round on 29 November 2022. We imported participants’ responses to MAXQDA Analytics Pro 2022, a qualitative analysis software,^35^ and ERV identified the text segments that met our EO response, empathic verbal expressions, and techniques coding categories. A second coder (ZD), who is an Associate Professor of Applied Linguistics, provided reliability coding. This involved independent coding of patient EOs in the 10 messages and reviewing the coding for all coach responses to messages 1 and 2. Any discrepancies and uncertainties were addressed through periodic meetings until a consensus was reached.

### Choosing the patient messages for the survey

We retrieved a sample of 236 patient messages from participants in the Irish and English arms of the Bump2Baby and Me study trial^36^ that were available at the time of the survey preparation (August 2022). The trial involves a tele-health coaching intervention designed to prevent gestational diabetes mellitus and facilitate postnatal weight loss in 800 women across Australia, Ireland, the United Kingdom, and Spain. We chose 10 messages according to 2 criteria: covering all the types of EOs (Table 1) and not being ambiguous, that is, including enough information to allow the HC to reply despite the lack of background information about the patient. These messages can be found in Appendix 2.

Textbox 1. Example message of the patient messages employed in the survey, showing clause separation and empathy opportunity (EO) labelling.

Hi (coach name).| im doing ok so far| did great step account on last month **(1. EO5i)**| but| just after I hit my 20week/5mnt mark. (2.1)| I feel extremely tired (2.2) **(2. EO1)**| and| struggling to get 10 thousand step per day **(3. EO2)**| and| partly I feel worn out **(4. EO1)**| and| a bit if pain cos womb is attached on the front of the belly wall **(5. EO7)**| and| I have constant “pulling/stretching” pain. **(6. EO2)**| less bread of course is still my coal l close list..| or now might I need to munch less cookies. **(7. EO4)**| its raining way too much for my liking **(8. EO7)**| and| not having no energy lately (9.1)| will I say effect my treats snacking and mood lifting with food. (9.2) **(9. EO7)**| I'll keep my goals atm the same (10.1)| just not to push my self more for something I can't accomplish (10.2)| and| not having then self let down moments (10.3) **(10. EO2)**| (participant name)

Where applicable, we separated the sentences used to express an EO into clauses (a group of words that comprises a subject and a verb^37^), which we called subEOs, for our unit of analysis. This is because subEOs represent different ideas within the same EO that the HC can address. For example, there are three clauses in EO5i ‘*Nausea and sickness has lessened* (EO5i.1)*| so| that has enabled me to eat more consciously* (EO5i.2)*| and| get back to healthy breakfasts* (EO5i.3)’. A HC may respond to all three or only one of them. We also created an additional subcategory for EO5 to differentiate explicit (EO5e) from implicit (EO5i) expressions of positive self-judgement. The distribution of subEOs represents the wider dataset and can be found in Appendix 3.

### Coding categories

For coding the coaches responses (n = 88) to the patient messages, we first mapped the BC, MBCT, and SDT techniques against each other, according to the overlaps identified by the work of Teixeira^16^ and Gillison.^12^ Furthermore, we classified the primary target of these techniques based on the three psychological needs associated to motivation according to SDT: autonomy, competence, and relatedness.^12^ By combining these frameworks, our goal was to integrate the optimal elements for eliciting behaviour change in an individual, with a specific focus on motivation (see Figure 1). We agreed on 28 techniques (Appendix 1) for our analysis based on their relevance to our study setting.

For the empathic response coding, we used Pounds’ classification of doctor's potential empathic verbal expressions.^25^ She differentiated two categories: Responding to patient cues and Eliciting patient's feelings and views (Table 2). We determined that an empathic response addressed an EO when the HC responses included a keyword that confirmed it. We included an additional coding category labelled ‘General’ for empathic responses that lacked a keyword but could be related to multiple EOs. To facilitate interpretation in the Results’ tables, we excluded the categories that had no counts in the dataset.

**Table 2.** Classification of doctors’ verbal empathic expressions, adapted from Pounds.^38^

	**Category**	**Description and examples**
**RESPONDING TO PATIENT CUES**	Expressing explicit **understanding or acknowledgement of patients’ feelings and views**	Formulations including verbs of acknowledgement (‘*I understand/see/realise/appreciate…*’), adjectival constructions expressing understanding (‘*It is clear/apparent… to me*’), or alternative formulations (‘It strikes me that…’, ‘I am aware/conscious that…’).
	**Sharing the patient's feelings or views** through expressions of agreement (‘emotive empathy’)	Shared feelings (‘*I would also… if I were you*’), shared feelings through interjection and intonation (‘*Oh no!*’), shared judgement (‘*Yes, your boss could have been more understanding*’), shared appreciation (‘*Yes, this is a difficult exercise*’)
	Expressing **acceptance** in response to patients’ explicit, implicit or potential negative or positive self-judgement	**Unconditional positive regard (or ‘praise’)** through: – Explicit expression of **positive judgement of the patient as a person** (‘*You are a fantastic mom*’) – Implicit expression of **positive judgement through explicit positive appreciation of the patient's actions or thoughts** (‘*It looks like you are making great progress*’) – Repetition or paraphrasing of patients’ words and avoidance of immediate countering statements or premature reassurance – Allowing patients to express their feelings and views fully through minimal responses, nodding, and avoidance of interruption.
		**Neutral support** (even when approval cannot be granted, withholding judgement): Explicit appreciation of the patient's behaviour, ideas or feelings in terms of their ‘**normality’ and ‘acceptability’** (‘*It is (completely) normal/not unusual/acceptable…to do/think/feel X*’, ‘*It is not (at all) surprising/crazy to do/think/feel X*’) Explicit expressions of judgement when **denying potential negative self-assessment by the patient** (‘*You are not odd, bad, crazy…for doing/thinking/feeling X…*’)
**ELICITING PATIENTS’FEELINGS AND VIEWS**	**Direct elicitation of patients’ feelings**	Enquiries about mental state (*‘How/what do/did you feel/think/expect?’*) or emotive behaviour (‘*How did/do you react?*’)
	**Indirect elicitation of patients’ feelings through:**	Questions about potentially negative/critical experiences (‘*Did you have a happy childhood?*’)
		Invitation of patients’ confirmation, rejection or clarification of interpreted affectual states (‘*You seemed concerned when I mentioned diabetes*’)
		Enquiries about the ‘emotive behaviour’ displayed (‘*Why are you crying?*’)
	**Direct elicitation of patients’ judgement**	‘*How are you finding your coach?*’
	**Indirect elicitation of patients’ judgement through:**	Enquiries about others or patient's behaviour (‘*How did your husband react?*’)
		Invitation of patients’ confirmation, rejection or clarification of interpreted views of others or themselves (‘*You seem very motivated to start your health programme’*)
	**Direct elicitation of patients’ appreciation**	‘*How would you rate your progress so far?*’
	**Indirect elicitation of patients’ appreciation through:**	Enquires about therapy or medication (‘*Would you like us to review your goals together?*’)
		Invitation of patients’ confirmation, rejection or clarification of interpreted view of things, events and actions (‘*You do not seem to find that book so useful*’)
	**Indirect elicitation of patients’ feelings and views** through formulations in which feelings and evaluations (potential or real) are attributed to **third parties** who might find/found themselves in circumstances similar to the patient's	‘*Many people in your position would be quite annoyed* [affect*]/would find this annoying [*appreciation*].’, ‘My sister went through something similar and she struggled to see the benefits of* [affect*]/to value* [appreciation] *that opportunity*’

## Results

### Coding results

 Table 3 shows an overview of the empathic responses (n = 121) coded in the HCs’ messages (n = 88), and their correlation with the patients’ subEOs. The missed subEOs (n = 15) are those that did not receive a reply from any HC, and these are mostly in implicit s (n = 12, 80% missed subEOs). While the overall response ratio is similar for implicit (89) and explicit (87) EOs, this value is higher for positive implicit EOs (97) and for negative explicit EOs (92).

**Table 3.** Overview of empathic responses.

	*Missed subEOs n (% missed EOs)*	*Response ratio*
***Explicit EOs (n = 27)***	3 (20)	89
*Positive (n = 2)*	1 (7)	50
*Negative (n = 25)*	2 (13)	**92**
***Implicit EOs (n = 94)***	12 (80)	87
*Positive (n = 29)*	1 (7)	**97**
*Negative (n = 65)*	11 (73)	83
***TOTAL (n = 121)***	**15** (**100)**	**85**

For each category, the Response Ratio was calculated by dividing the number of subEOs by the number of not missed subEOs. EO: empathy opportunity.

Table 4 shows the EO categories distribution, the missed subEOs, the HC responses, and the response ratios. In our study, HCs missed 14.3% (n = 15) of subEOs. These were mostly implicit and negative, EO7 (n = 6, 4.7%) and EO4 (n = 4, 3.1%). Although most of the HC responses addressed the implicit positive category EO5i, the highest response ratio was for its explicit form EO5e (39). The lowest response ratios were for EO3 (18) within the explicit EOs and overall, and for EO7^39^ among the implicit categories.

**Table 4.** Missed subEOs, coach responses count, and calculated response ratio.

*EO categories*	*Missed, n (% subEOS)*	*Coach responses, n (%)*	Response ratio (responses/subEOs*9)
***EXPLICIT EOS (n = 27)***	*3* (*5.5)*	*56* (*20.4)*	*23*
***Positive***			
***EO5e – Self-judgement (n = 2)***	1 (0.8)	7 (2.6)	**39**
***Negative***			
***EO1 – Feelings (n = 9)***	1 (0.8)	**20** (**7.3)**	25
***EO3 – Judgement (others or self) (n = 7)***	1 (0.8)	11 (4.0)	18
***EO6 – Appreciation (things, events, actions) (n = 9)***	0	18 (6.6)	22
***IMPLICIT EOs (n = 94)***	*12* (*6.3)*	*203* (*74.1)*	*24*
***Positive***			
***EO5i – Self-judgement (n = 29)***	1 (0.8)	**65** (**23.7)**	25
***Negative***			
***EO2 – Feelings (n = 20)***	1 (0.8)	52 (19.0)	**29**
***EO4 – Judgement (others or self) (n = 15)***	**4** (**3.1)**	32 (11.7)	24
***EO7 – Appreciation (things, events, actions) (n = 30)***	**6** (**4.7)**	54 (19.7)	20
***GENERAL REPLY (n = 6)***	*N/A*	*15* (*5.5)*	
***TOTAL (n = 127)***	**15** (**14.3)**	**274**	

EO: empathy opportunity.

Table 5 shows the techniques found in the empathic responses. We find the highest use of techniques overall for the MBCTs category. The most common is Acknowledge/respect patient's feelings/views (MBCT8, ‘*Well done on substituting unhealthy snacks for fruits!! That's a great idea*’), which promotes autonomy, especially in response to implicit statements such as expressions of positive self-judgement (EO5i, ‘*I have engaged in a 100k in 30 days for charity with work.*’) (n = 47, 31.1% MBCTs). However, when an implicit negative appreciation is made by the patient (EO7, ‘*My water intake has dropped*’), the coaches show unconditional regard (MBCT10, ‘*I totally get life gets in the way sometimes and it makes it harder to implement changes.*’) (n = 12, 7.9% MBCTs), which targets relatedness. This is also the most used category in response to the expression of explicit statements such as negative feelings (EO1, ‘*I’m just wrecked*’) (n = 5, 3.3% MBCTs) and negative judgements (EO3, ‘*Kids are quite demanding*’) (n = 4, 1.3% MBCTs), and when the coach response is general (‘*Oh my goodness, it does sound as though you have a lot going on at the moment!*’) (n = 7, 4.6% MBCTs).

**Table 5.** Techniques coded in the HC responses.

		EXPLICIT				IMPLICIT				General, n (%)
		EO1-E negative feelings, n (%)	EO3-E negative judgement, n (%)	EO5e positive self-judgement, n (%)	EO6-E negative appreciation, n (%)	EO2-I negative feelings, n (%)	EO4-I negative judgement, n (%)	EO5i positive self-judgement, n (%)	EO7-I negative appreciation, n (%)	
**DOMAIN**	**TECHNIQUES (n = 300)**	**17** (**5.7)**	**11** (**3.7)**	**8** (**2.7)**	**20** (**6.7)**	**56** (**18.7)**	**54** (**18.0)**	**75** (**25.0)**	**46** (**15.3)**	**13** (**4.3)**
	**Motivational Behaviour Change Techniques (MBCTs) (n = 151)**	8 (5.3)	7 (4.6)	4 (2.6)	6 (4.0)	21 (13.9)	18 (11.9)	54 (35.8)	22 (14.6)	11 (7.3)
***Relatedness-support***	MBCT 8 Acknowledge/respect perspectives/feelings	3 (2.0)	3 (2.0)	3 (2.0)	2 (1.3)	**13** (**8.6)**	**11** (**7.3)**	**47** (**31.1)**	**8** (**5.3)**	4 (2.6)
	MBCT 9 Encourage asking of questions						3 (2.0)			
	MBCT 10 Show unconditional regard	**5** (**3.3)**	**4** (**2.6)**	1 (0.7)	2 (1.3)	**8** (**5.3)**	4 (2.6)	1 (0.7)	**12** (**7.9)**	**7** (**4.6)**
	MBCT 11 Demonstrate interest in the client				2 (1.3)			6 (4.0)	2 (1.3)	
	**Behaviour Change Techniques (BCTs) (n = 114)**	5 (4.4)	2 (1.8)	4 (3.5)	**11** (**9.6)**	**29** (**25.4)**	**26** (**22.8)**	19 (16.7)	16 (14.0)	2 (1.8)
***Goals and planning***	BCT 1.1 Goal-setting			1 (0.9)			1 (0.9)			
	BCT 1.2 Problem-solving	1 (0.9)			**4** (**1.3)**	**6** (**5.3)**	**4** (**3.5)**	**4** (**3.5)**	**4** (**3.5)**	
	BCT 1.4 Action planning		1 (0.9)	1 (0.9)	**4** (**1.3)**		**5** (**4.4)**		**3** (**2.6)**	
	BCT 1.5 Review behaviour goal(s)		1 (0.9)			**10** (**8.8)**		1 (0.9)	1 (0.9)	1 (0.9)
***Feedback and monitoring***	BCT 2.3 Self-monitoring of behaviour				1 (0.9)	1 (0.9)	1 (0.9)	1 (0.9)	1 (0.9)	
***Social support***	BCT 3.1 Social support	1 (0.9)				2 (1.8)	2 (1.8)		**3** (**2.6)**	1 (0.9)
***Shaping knowledge***	BCT 4.2 Information about antecedents	1 (0.9)			1 (0.9)			2 (1.8)		
***Natural consequences***	BCT 5.1 Information about health consequences					2 (1.8)	2 (1.8)	1 (0.9)	2 (1.8)	
	BCT 5.4 Monitoring of emotional consequences	1 (0.9)						2 (1.8)		
	BCT 5.6 Information about emotional consequences			1 (0.9)		1 (0.9)		2 (1.8)	1 (0.9)	
***Associations***	BCT 7.1 Prompts/cues							2 (1.8)		
	BCT 8.2 Behaviour substitution						**4** (**3.5)**			
	BCT 8.3 Habit formation						1 (0.9)			
	BCT 9.1 Credible source	1 (0.9)		1 (0.9)	1 (0.9)	**5** (**4.4)**	2 (1.8)	**4** (**3.5)**	1 (0.9)	
	BCT 12.1 Restructuring physical environment					1 (0.9)	**4** (**3.5)**			
	BCT 13.2 Framing/reframing					1 (0.9)				
	**Self Determination Techniques (SDTs) (n = 35)**	4 (11.4)	2 (5.7)		3 (8.6)	6 (17.1)	10 (28.6)	2 (5.7)	8 (22.9)	
	SDT 4 Use of non-controlling language				3 (8.6)	4 (11.4)	**9** (**25.7)**	1 (2.9)	4 (11.4)	
	SDT 5 Intrinsic goal orientation	**4** (**11.4)**	2 (5.7)			2 (5.7)	1 (2.9)	1 (2.9)	4 (11.4)	

Domains are shown for MBCTs and BCTs according to Teixeira's^18^ and Michie's^10^ classification, respectively. Percentages are expressed for total MBCTs, BCTs, and SDTs categories, separately. The empty cells represent a 0 value.

**Figure 2.**
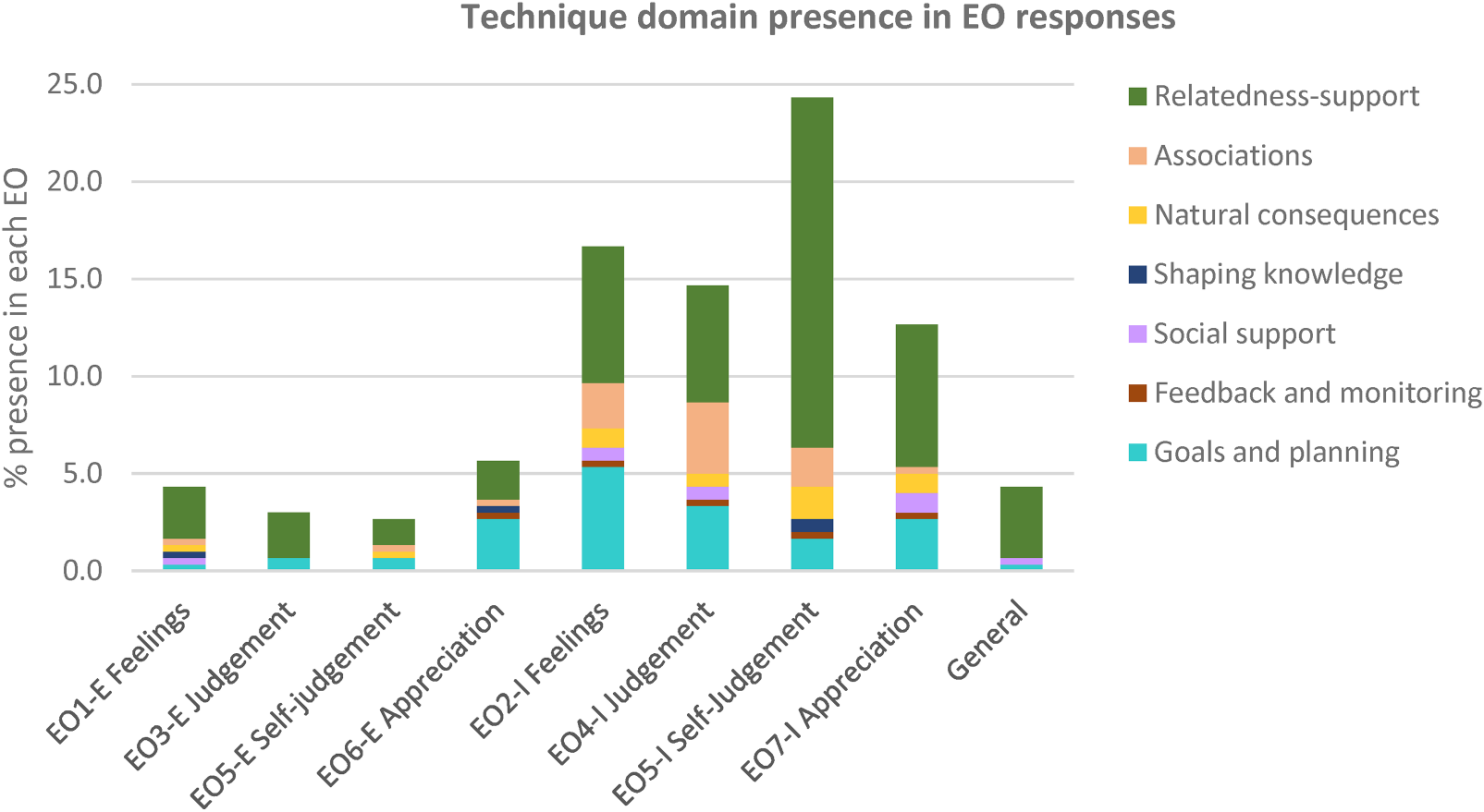
Technique domain presence for each empathy opportunity response. E: explicit; EO: empathy opportunity; I: implicit.

Figure 2 offers a summary of the distribution of technique domains in response to EOs. Implicit EOs are more frequently addressed than explicit ones, with a predominant use of Relatedness-support techniques (MBCTs), followed by Goals and Planning and Associations BCTs. Although coaches did not use BCTs extensively, we found some common responses to implicit EOs. When there are implicit negative feelings being expressed (EO2, ‘*Don’t know how to manage this.*’) (n = 29, 25.4% BCTs), the most used techniques are Competence-promoting BCTs such as Review behaviour goals (BCT 1.5, ‘*You can always bring your steps goal down until you are pain-free*’) (n = 10, 8.8% BCTs) and Problem-solving (BCT 1.2*,* ‘*Maybe we can find options to do at home while the weather is not great*’) (n = 6, 5.3% BCTs), as well as Sharing information from a credible source (BCT 9.1, ‘*I have attached the guidelines on physical activity in pregnancy so that you can compare them to what you are doing.*’) (n = 5, 4.4% BCTs).

When there might be a negative expression of judgement about others or self (EO4, ‘*Never managed to start up yoga or other exercise beyond daily life needs*’), there is a slight tendency to use Action planning (BCT 1.4, ‘*Do you ever plan your meals for the week? (…) I've attached a weekly meal planning template for you to print out as a guide.*’) (n = 5, 4.4% BCTs) to promote autonomy, and an identical count for Problem-solving (BCT 1.2), targeting competence, as well as Behaviour substitution (BCT 8.2, ‘*Perhaps you could aim to have a supply of nutritious snacks that you would be happy to munch on when the need for a snack hits?*’) and Restructuring physical environment (BCT 12.1, ‘*Putting the cookies away after you eat them, and in a higher up cupboard can also make this easier!*’) (n = 4, 3.5% BCTs each).

Similarly, when the patient makes an implicit negative appreciation (EO7), the coach turns to Problem-solving (BCT 1.2) (n = 4, 3.5% BCTs), as well as Action planning (BCT 1.4) and Social support (BCT 3.1, ‘*Is there a family member, friend or neighbour that can help out perhaps?*’) (n = 3, 2.6% BCTs each). Positive self-judgement statements from the patient (EO5) are typically addressed through Action planning (BCT 1.4) and providing information from a Credible Source (BCT 9.1) (n = 4, 3.5% BCTs each). Regarding explicit EOs, BCTs are mostly used to reply to Explicit negative appreciation (EO6) (‘*Breastfeeding mega demanding and sore*’) (n = 11, 9.6 BCTs) again with the combination of BCTs Problem solving (1.2) and Action planning (1.4) (n = 4, 3.5% BCTs each), similarly to its implicit version or Implicit negative appreciation (EO7). In the SDT category, the techniques that we identified promote autonomy mostly through the Use of non-controlling language (SDT4, ‘*And remember that myself and the app are here to help you in ways that suit your needs, so if you do not want to log behaviours that is ok.*’) in response to a Negative expression about others or self (EO4, n = 9, 25.7% SDTs) and Intrinsic goal orientation (SDT 5, ‘*Can you think of anything to help your mental wellbeing in this time?*’) to reply to Explicit negative feelings (EO1, n = 4, 11.4% SDTs).

 Table 6 shows the empathic analysis’ results. For the ‘Responding to patient cues’ empathic category (n = 189, 67% empathy appraisal categories), we find the highest count of empathic responses for the ‘Implicit positive expression of self-judgement’ category (EO5-i, n = 57, 30.2% Responding to patient cues) mostly through ‘Acceptance’ in the form of ‘Unconditional positive regard’ and expressed as ‘Praise’ (‘*Amazing exercise! That's fabulous to see you have reached your exercise goals.*’) (n = 49, 25.9% Responding to patient cues), which HCs also use to address ‘Implicit expressions of feelings’ or EO2 (n = 11, 5.8% Responding to patient cues) (‘*Thank you for sharing your challenge with the app.*’). In contrast, empathic responses to ‘Implicit expressions of appreciation’ (EO7, n = 36, 19%) are spread among ‘Agreement’ by ‘Sharing feelings and views’ (n = 11, 5.8% Responding to patient cues) (‘*I know unfortunately the weather isn’t always great and not something that we can control and so it can be hard to rely on for energy.*’), ‘Acceptance’ in the form of ‘Neutral support’ by ‘Normalising through an explicit appreciation’ (n = 10, 5.3% Responding to patient cues) (‘*Everything is always so much harder when you aren't getting enough sleep.*’), and ‘Explicit understanding or acknowledgement of patients’ feelings and views’ (‘*I understand your reluctance around not adding in new, varied forms of exercise just yet*’) (n = 11, 5.8% Responding to patient cues). When the empathic response is ‘General’, coaches tend to express ‘Acceptance’ in the form of ‘Unconditional positive regard’ through a repetition of the patients’ statement (‘*Yes writing your food down can create some natural feedback.*’) (n = 7, 3.7% Responding to patient cues).

**Table 6.** Empathy appraisal categories coded in the HC responses.

	EXPLICIT				IMPLICIT				General
	EO1-E feelings, n (%)	EO3-E judgement, n (%)	EO5-e self-judgement, n (%)	EO6-E appreciation, n (%)	EO2-I feelings, n (%)	EO4-I judgement, n (%)	EO5-i self-judgement, n (%)	EO7-I appreciation, n (%)	
**RESPONDING TO PATIENT CUES (n = 189, 67%)**	**16** (**8.5)**	**10** (**5.3)**	**4** (**2.1)**	**3** (**1.6)**	**31** (**16.4)**	**20** (**10.6)**	**57** (**30.2)**	**36** (**19.0)**	**12** (**6.3)**
**Acceptance**									
**Unconditional positive regard**									
**Repetition – No counter**						1 (0.5)	1 (0.5)	1 (0.5)	**7** (**3.7)**
**Praise - Expl/impl positive judgement**		2 (1.1)	2 (1.1)		**11** (**5.8)**	**7** (**3.7)**	**49** (**25.9)**	2 (1.1)	
**Neutral support**									
**Normalising (not explicit appreciation)**					1 (0.5)			1 (0.5)	
**Explicit judgement**							1 (0.5)		1 (0.5)
**Normalising (explicit appreciation)**	**9** (**4.8)**	**5** (**2.6)**	1 (0.5)		8 (4.2)	7 (3.7)	5 (2.6)	**10** (**5.3)**	1 (0.5)
**Sharing feelings and views (agreement)**	3 (1.6)		1 (0.5)	3 (1.6)	2 (1.1)	2 (1.1)	1 (0.5)	**11** (**5.8)**	3 (1.6)
**Explicit understanding or acknowledgement of patients’ feelings and views**	4 (2.1)	3 (1.6)			9 (4.8)	3 (1.6)		**10** (**5.3)**	
**ELICITING PATIENT FEELINGS AND VIEWS (n = 93, 33%)**	**8** (**8.6)**	**3** (**3.2)**	**4** (**4.3)**	**11** (**11.8)**	**18** (**19.4)**	**18** (**19.4)**	**14** (**15.1)**	**14** (**15.1)**	**3** (**3.2)**
**Appreciation**									
**Indirect**									
Patients’ confirmation, rejection or clarification	1 (1.1)						3 (3.2)		
Therapy or medication (coaching programme)	**4** (**4.3)**	3 (3.2)	2 (2.2)	**9** (**9.7)**	**14** (**15.1)**	**15** (**16.1)**	7 (7.5)	**11** (**11.8)**	1 (1.1)
**Direct**			1 (1.1)						
**Judgement**									
**Indirect: About others or patient's behaviour**						1 (1.1)			
**Feelings**									
**Indirect**									
Enquiries about the emotive behaviour displayed				1 (1.1)					
Invitation of patients’ confirmation, rejection or clarification	1 (1.1)		1 (1.1)	1 (1.1)	3 (3.2)			3 (3.2)	**2** (**2.2)**
Questions about potentially negative/critical experiences	1 (1.1)								
**Direct**	1 (1.1)				1 (1.1)	1 (1.1)	4 (4.3)		
**Feelings and views - indirect (3rd parties)**						1 (1.1)			

The empty cells represent a 0 value. HC: health coaches.

As observed in prior tables, we find a higher response rate for implicit than explicit statements in the ‘Eliciting patient feelings and views’ category (n = 93, 33% empathy appraisal categories). In this category, most coded responses belong to the ‘Indirect appreciation about therapy or medication’ category (in our intervention, lifestyle goals and behaviours) (‘*Think about the long-term aim of focusing on these smaller habits- can you think of anything to help your mental wellbeing in this time?*’) in response to implicit expressions of negative judgements (EO4) (n = 15, 16.1% Eliciting patient feelings and views), negative feelings (EO2) (n = 14, 15.1% Eliciting patient feelings and views), and negative appreciations (EO7, n = 11, 11.8% Eliciting patient feelings and views). However, there is also a relatively high count of the same response category (Indirect appreciation about therapy or medication) to explicit negative expressions of appreciations (EO6, n = 9, 9.7%) and feelings (EO1, n = 4, 4.3%, respectively). When the coaches chose a ‘General’ response to elicit patient feelings and views, they mostly invited the patient to confirm, reject or clarify their feelings indirectly (‘*It sounds like you are happy with the way things are going at the moment*’) (n = 2, 2.2%).

## Discussion

### Principal findings

The high proportion of negative and implicit EOs in our coding scheme affected the coach responses’ distribution across coding categories. For this reason, the response ratio was a better reflection of the overall coach behaviour. Our findings reveal that while coach responses align with their training, they also show a degree of variability and diverse approaches to the same EO, often with the same objective or primary target. This variability may be influenced by their background and interpersonal skills, which shape their empathetic attitude. The use of different approaches raises the question of whether the use of specific techniques truly matters when the HCs share a common goal. While these differences are significant at the technique level, they might not substantially impact fidelity assessment when evaluating the programme's overall effectiveness.

We found the highest response ratio for explicit expressions of positive self-judgement (‘*I am motivated to do as much as I can regarding healthy choices*’) and the lowest response ratio for explicit negative judgements about others or self (‘*Kids are quite demanding*’). This finding could be attributed to the coach's avoidance of negative feedback and emphasis on positive reinforcement. Another contributing aspect could be that HCs lacked context and familiarity with the patients, potentially hindering their response to negative cues in the absence of an established patient–provider relationship. Nonetheless, due to the limited number of instances in our dataset (n = 4 and n = 2 for EO3 and EO5e, respectively), we need further research to determine the appropriate empathic responses within these categories.

Coach responses to other explicit EOs were spread across categories, with the highest counts for addressing expressions of negative appreciations (EO6) and negative feelings (EO1) by eliciting patients’ appreciation on the coaching programme or normalising their feelings, respectively (Table 7).

**Table 7.** Examples of the most frequently addressed EOs and their HC responses.

**Examples**	**Coding**
**P: *The next two weeks are extremely busy at work.***	**EO6:** Explicit expression of negative appreciation
**HC: *After lunch, perhaps you might fit a walk into your busy agenda.***	**Eliciting patient feelings and views** indirectly about the coaching programme or their goals
**P: *I am just wrecked.***	**EO1**: Explicit expression of negative feelings
**HC: *It is hard to focus on new things when we have a lot of stress going on.***	**Responding to patient cues** through normalising with an explicit appreciation

EO: empathy opportunity; HC: health coaches.

In Pounds’ framework, ‘Normalizing’ serves as a form of ‘neutral support’, validating negative emotions to alleviate their distress and foster acceptance and support. This normalisation can also create a space where patients do not feel isolated by recognising that their experiences are understood and shared by others. Our assumptions are supported by the use of relatedness and autonomy-promoting techniques such as showing unconditional regard (MBCT10) and intrinsic goal orientation (SDT5), which illustrate the coaching methodology's alignment with the SDT principles. Other interesting responses to explicit negative appreciation and feelings consisted of competence and autonomy-promoting Goals and planning BCTs, as well as eliciting patients’ appreciation on the coaching programme, which denotes interest in the patient and allows intervention tailoring. This approach fosters rapport and, consequently, relatedness. Making sure that goals are meaningful and well received by the patient promotes BC and prevents loss of motivation and negative emotions.^40^ These findings correspond to the principles of GROW's model^34^ used by Liva coaches to promote autonomy and guide the patient in developing their own conclusions. In this context, future research work examining how Pounds’ categories serve the three psychological needs could enhance our understanding of the relationship between BC and empathy.

Other notable findings showed that when patients expressed a judgement, HC responses included codes from both empathy appraisal and BC categories that often overlapped or were next to each other. For example, a patient's expression of either a negative or a positive judgement about themselves was often seen by the HC as an opportunity to Praise and Acknowledge their perspective/feelings in the form of awareness (Table 8). This is an interesting finding since some studies on doctors’ communication skills highlight the frequency of missed empathic or praise opportunities.^28,38^ We could attribute our results to the person-centered training that our HCs received, proving its relevance to any HCP. Moreover, a further study of these overlapping categories could improve the coding process and ultimately allow for the creation of a simplified coding guide.

**Table 8.** Examples of HC approaches to patients’ expression of judgments.

**Patient**		**Coach**	
**EO**	**Coding**	**Response**	**Coding**
***… or now might I need to munch less cookies.***	**EO4:** Implicit negative expression of judgment about others or self	*Well done on thinking on cutting down on sugary things and bread, you're thinking of the best options for your health, congratulations!*	Responding to patient cues with **Praise** and **MBCT8:** Acknowledge perspective/feelings in the form of awareness
***I did a great step account on last month.***	**EO5i:** Implicit positive expression of self-judgment	*I am so glad to hear you celebrating your step count success for last month. Celebrating our wins is such an important part of behaviour change.*	

EO: empathy opportunity; HC: health coaches; MBCT: motivational behaviour change technique.

Given the complexity of human communication, it is expected that HCs employ different strategies for addressing each EO. For example, responses to implicit expressions of negative feelings involved up to six different techniques, including Relatedness-Support MBCTs, Goals and planning BCTs, Associations BCTs, and one SDT. Though these techniques collectively address the three psychological needs, the selection of one over another by HC signifies their identification of the most crucial need to be addressed in the given situation. However, it is important to approach conclusions from our study with caution, as the small sample size absence of a coach–patient relationship may have led to a potentially random selection of these techniques.

Overall, our findings suggest that certain codes may combine more effectively in written interactions for a theoretically optimal response to patient cues. Nevertheless, empirical research is necessary to examine the practical implications of these combinations. Despite inevitable differences among HC responses, understanding the extent to which that variability affects the effective implementation of a behaviour change programme is essential. As a starting point, our article establishes the foundation for a fidelity assessment framework that can support future asynchronous health interactions research. In addition to refining the proposed categories with larger samples, upcoming studies should connect these categories to both short-term and long-term patient outcomes. This will help identify optimal responses and, ultimately, enhance provider training for improved patient–provider communication and intervention outcomes.

### Strengths and limitations

Our study design presents both strengths and limitations. We selected 10 patient messages that represented all the EO categories, and the participation of 11 HC ensured a diverse sample that displayed the variability of responses to the same message. The two-round survey facilitated a systematic examination of these responses, providing detailed and relevant data for analysis with a well-defined coding framework. However, we acknowledge some limitations. The number of survey messages and participants was relatively small, and the sample was limited to English-speaking HCs, which may restrict the generalisability of our results to other patient populations and all HCs. In some cases, the limited number of EOs and empathic responses posed challenges to drawing conclusions. Furthermore, our survey presented a simulated interaction that differs from the Bump2Baby and Me trial. In this context, messages lacked patient-specific background information, and the HC did not have an established relationship with the patients, which could have influenced their responses. In real-world coaching scenarios, HCs have access to patient-specific details and develop a coaching relationship over time, allowing them to personalise responses more effectively. Additionally, the HCs’ participation in the study could have introduced an observer bias. Although we anonymised their responses, their participation awareness might have affected their behaviour. Additionally, the use of the same messages in both rounds might have influenced Round 2 responses. Considering these strengths and limitations, we recommend cautious interpretation of our findings and encourage further research with larger and more diverse samples to validate and extend our conclusions.

### Conclusion

Our study provides valuable insights into HCs’ responses to different EO categories. While the high proportion of negative and implicit EOs influenced the distribution of HCs’ empathic responses’, which aligned with their training. We observed the highest response ratio for explicit expressions of positive self-judgement and the lowest response ratio for explicit negative judgement about others or oneself, which may be attributed to HCs’ tendency to avoid negative feedback and emphasise positive reinforcement. The implicit expressions of negative feelings and negative judgement were addressed with multiple techniques, often related to the domains of Relatedness-support, Goals and planning, and Associations. The overlap and co-occurrence of codes across coding categories show the complexity of human communication and the various approaches HCs may employ to address the same EOs. This variability raises questions about the need for specific techniques for achieving the same objective. Assessing the impact of this variability on patient outcomes could offer valuable insights for BC programme implementation and fidelity. Furthermore, there is a need for further research to explore empathic responses within EO categories with limited instances, examine combinations of techniques within the same response, and understand how Pounds’ categories address the psychological needs of autonomy, competence, and relatedness.^41^

## Supplemental Material

sj-docx-1-dhj-10.1177_20552076231225889 - Supplemental material for Digital empathy in behaviour change interventions: A survey study on health coach responses to patient cues Supplemental material, sj-docx-1-dhj-10.1177_20552076231225889 for Digital empathy in behaviour change interventions: A survey study on health coach responses to patient cues by E Rey Velasco, Z Demjén, TC Skinner and on behalf of the Impact Diabetes B2B Collaboration Group in DIGITAL HEALTH
