# Supplementary material for: Digital empathy in behaviour change interventions: A survey study on health coach responses to patient cues
Source: Digit Health. 2024 Mar 15;10:20552076231225889. doi: 10.1177/20552076231225889 (PMC10962034; doi:10.1177/20552076231225889)
Supplement: sj-docx-1-dhj-10.1177_20552076231225889 - Supplemental material for Digital empathy in behaviour change interventions: A survey study on health coach responses to patient cues [file sj-docx-1-dhj-10.1177_20552076231225889.docx]

# APPENDICES

## Appendix 1: Overview of techniques mapping

| Grouping and BCTs | MBCTs | SDTs | Primary target |
| --- | --- | --- | --- |
| 1.1 Goal-setting (goal) | MBCT 17. Explore barriers and obstacles | SDT6: Structure | Autonomy |
| 1.2. Problem-solving | MBCT 15. Ask permission to provide information or give advice | SDT14: Barrier identification | Competence |
| 1.3. Goal-setting (outcome) |  | SDT6: Structure | Autonomy |
| 1.4. Action planning | MBCT 19. Assist in setting optimal challenge | SDT6: Structure | Autonomy |
| 1.5. Review behaviour goal(s) |  | SDT12: Provide informational feedback | Competence |
| 1.6. Discrepancy between current behaviour and goal |  |  |  |
| 1.7. Review outcome goal(s) |  | SDT12: Provide informational feedback | Competence |
| 1.9. Commitment |  |  |  |
| 2.3. Self-monitoring of behaviour | MBCT 18. Clarify expectations | SDT12: Provide informational feedback | Competence |
| 2.4. Self-monitoring of outcome(s) of behaviour |  | SDT12: Provide informational feedback | Competence |
| 2.6. Biofeedback |  | SDT12: Provide informational feedback | Competence |
| 3.1. Social support (unspecified) | MBCT 14. Use attentive, reflective listening | SDT17: Encourage social support seeking | Relatedness |
| 4.2. Information about antecedents |  | SDT3: Provide a rationale SDT13. Provide information | Autonomy Competence |
| 4.3. Re-attribution |  |  |  |
| 5.1. Information about health consequences |  | SDT3: Provide a rationale SDT13. Provide information | Autonomy Competence |
| 5.4. Monitoring of emotional consequences |  |  |  |
| 5.6. Information about emotional consequences |  | SDT3: Provide a rationale SDT13. Provide information | Autonomy Competence |
| 7.1. Prompts/cues |  |  |  |
| 8.2. Behaviour substitution |  |  |  |
| 8.3. Habit formation |  |  |  |
| 8.4. Habit reversal |  |  |  |
| 8.6. Generalisation of target behaviour |  |  |  |
| 8.7. Graded tasks | MBCT 17. Explore barriers and obstacles | SDT11: Provide optimal challenge | Competence |
| 9.1. Credible source |  |  |  |
| 11.2. Reduce negative emotions |  |  |  |
| 11.3. Conserving mental resources |  |  |  |
| 12.1. Restructuring the physical environment |  |  |  |
| 12.2. Restructuring the social environment |  | SDT7: Emphasise responsibility | Autonomy |
| 12.4. Distraction |  |  |  |
| 12.6. Body changes |  |  |  |
| 13.1. Identification of self as role model |  |  |  |
| 13.2. Framing/reframing |  |  |  |
| 15.1. Verbal persuasion about capability |  | SDT15: Provide support and encouragement | Competence |
| 15.4. Self-talk |  |  |  |
|  | **MBCT 8. Acknowledge and respect perspectives and feelings** |  |  |
|  | **MBCT 9. Encourage asking of questions** |  |  |
|  | **MBCT 10. Show unconditional regard** |  |  |
|  | **MBCT 11. Demonstrate/show interest in the person** |  |  |
|  | **MBCT 12. Use empathic listening** |  |  |
|  |  | **SDT4: Use of non-controlling language** | Autonomy |
|  |  | **SDT5: Intrinsic goal orientation** | Autonomy |
|  |  | **SDT10: Task climate** | Competence |

## APPENDIX 2: Survey messages

| **Message 1** | HI (coach name). \|sorry for not answering sooner.\|seems to time files (1.1)\| and\| I get nothing done (1.2) **(1. EO4)**\| and \|every day seems to have less and less energy to do anything. **(2. EO7)**\| its been 3 days raining **(3. EO7)**\| and \|I have had very little sleep (4.1)\| because of\| my daughter bloods are very unsuitable at night time. (4.2) **(4. EO6)**\| can't walk anymore that much **(5. EO7)**\| seems its pulling my belly muscles (6.1)\| and\| it's really hurts (6.2) **(6. EO7)**\| (dr said its attached to the front wall) \| a quess that's why the pain\| and \|I feel already so big (emojis). **(7. EO2)**\| I'm trying to eat lots of fruits. **(8. EO5i)**\| not being healthy as I wish (9.1)\| cos\| 5 days a week I'm to one who is cooking and looking after the house (9.2) **(9. EO4)**\| (husband does 12h shifts 5-6days a week). **(10. EO7)** \| I'm just wrecked **(11. EO1)**\| and \|if I feel so I have a cookie or chocolate or 2-4...\| hopefully wheater improves and so my energy levels\| and\| being a bit more healthier then atm.\| and \|no I didn't do the snacks. **(12. EO4)**\| I cook every day 3 meals for fam of 5 (13.1) \| and \|atm I do not have that energy to do extra at any level (13.2) **(13. EO2)**\| sorry. \| thank you for your messages\| and\| talk to you soon (participant name) \| |
| --- | --- |
| **Message 2** | \|The next two weeks are extremely busy in work with the last two teaching weeks of term. **(1. EO6)**\| It should hopefully be less hectic once it gets to corrections.\|Life is very busy and stressful at the moment, **(2. EO6)** \|so\| I am only focusing on making sure I get the supplements every day (3.1)\| and\| trying not to eat too much chocolate for the next two weeks. (3.2) **(3. EO5i)**\| I've been trying to add some fruit, **(4. EO4)**\| but\| I'm still eating loads of veggies, **(5. EO5i)**\| so\| I'm not overly stressing about that one.\| I know exercise helps with all this,\| but\| I don't have the time or headspace to fit it in at the moment, especially not something new. **(6. EO2)**\| I will look at the stretches and set a new goal once I have survived this fortnight!\| |
| **Message 3** | hi (coach name),\| Thanks for your videos ,\| only seeing the last 2 now.\| Yes all is fine with the goals.\| However you are right,\| logging them and engaging in app is not top priority at the moment. **(1. EO7)**\| I am happy with the goals I've set,\| breakfast ,water and walking is being achieved at my pace. **(2. EO5i)**\| I am motivated to do as much as I can regarding healthy choices **(3. EO5e)**\| but\| also listening to my body when I need rest and a cup of tea. **(4. EO5i)**\| Nausea and sickness has lessened in the last 2 weeks (5.1)\| so\| that has enabled me to eat more consciously (5.2)\| and\| get back to some healthy breakfasts (like yoghurt , fruit and seeds etc) (5.3) **(5. EO5i)**\| Walking in the evening is still sporadic **(6. EO7)**\| but\| I am enjoying it\| and\| have engaged in a 100k in 30 days for charity with work. **(7. EO5i)**\| again I am doing this when I feel I can and at my own pace.\| i am happy with my diet and behaviour right now\| and\| cravings are not an issue actually. **(8. EO5i)**\| I find my blood sugar to be stable too. **(9. EO5i)**\| Unfortunately I cannot promise more engagement with App, (10.1)\| I will log when I think of it. (10.2) **(10. EO7)**\| hope thats okay (emojis) \| thanks again, (participant name) |
| **Message 4** | Hi (coach name).\| im doing ok so far\| did great step account on last month **(1. EO5i)**\| but\| just after I hit my 20week/5mnt mark. (2.1)\| I feel extremely tired (2.2) **(2. EO1)**\| and\| struggling to get 10 thousand step per day **(3. EO2)**\| and\| partly I feel worn out **(4. EO1)**\| and\| a bit if pain cos womb is attached on the front of the belly wall **(5. EO7)**\| and\| I have constant "pulling/stretching" pain. **(6. EO2)**\| less bread of course is still my coal l close list..\| or now might I need to munch less cookies. **(7. EO4)**\| its raining way too much for my liking **(8. EO7)**\| and\| not having no energy lately (9.1)\| will I say effect my treats snacking and mood lifting with food. (9.2) **(9. EO7)**\| I'll keep my goals atm the same (10.1)\| just not to push my self more for something I can't accomplish (10.2)\| and\| not having then self let down moments (10.3) **(10. EO2)**\| (participant name) |
| **Message 5** | Hi (coach name),\| I did a recording (1.1)\| but\| I am not too sure where it saved (1.2)\| so\| I stick with writing a little paragraph. (1.3) **(1. EO7)**\| Last week was a great week for exercise.\| I reached my 7 hrs a week exercise by doing my hour walk Monday to Friday **(2. EO5i)**\| and\| I started my exercise bike Friday (3.1)\| and\| do that for 20 mins in the morning. (3.2) **(3. EO5i)**\| Last thursday I became really fizzy whilst on my walk and sweating. **(4. EO7)**\| A weird sensation that came over me for about 10 mins. **(5. EO2)**\| Perhaps my stomach was empty (6.1)\| or\| was just over tired. (6.2) **(6. EO7)**\| My diet has not been great, **(7. EO7)**\| although\| Ive been drinking my water, **(8. EO5i)**\| food intake and treats have been a weakness, (9.1)\| especially over the bank holiday weekend being out for picnics and a birthday party (9.2) **(9. EO7)**.\| The tips on physical activity is good.\| I actually do a good bit of gardening and cleaning (10.1)\| so\| I should incorporate that into my exercise. (10.2) **(10. EO5i)** I literally do not stop at home from 6.30 am intill 7pm **(11. EO2)**\| Kids are quite demanding **(12. EO3)** (emoji) |
| **Message 6** | Hello (coach name) good morning.\| My week was great, tnks God (emojis)\| the only problem was that I got so angry with my husband during the week. **(1. EO1)**\| Is it normal to get so angry during pregnancy?\| I hope you had a good week too.\| Thank you very much for the link,\| I gonna try to make something (emoji)\| and\| to contact the clinic.\| I have to take a vaccine on Wednesday\| and\| I can ask to my GP if he got my results.\| For any reason i ate much better during last week. **(2. EO5i)**\| I'll start to write down my all of my food,\| as you said.\| I think is going to be much better to understand what's happening. |
| **Message 7** | Hi (coach name),\| I was having a rough week last week. **(1. EO2)**\| Busy with work (2.1) \| and also I wasunwell some days. (2.2) **(2. EO1)**\| I feel better now.\| And I will continue updating my daily goals. **(3. EO5i)**\| Thanks for the support.\| I don't us my mobile phone regularly.\| I am not familiar with apps and all. **(4. EO4)**\| I have to depend on my husband for technical things. **(5. EO4)**\| That's why I am not up to date.\| And moreover my smart phone is an old one. **(6. EO6)**\| That's why I can't show the steps in the mobile.\| Thank you (participant name) |
| **Message 8** | Hi (coach name)\| Thank you!\| Yes baby boy born Wednesday,\| a lovely calm elective section\| and\| only 31 hours in hospital after.\| I am useless (1.1)\| sorry\| missed all of these messages and most from family and friends (emojis) (1.2) **(1. EO3)**\| Definitely did too much at once with a pretty non flexible deadline! **(2. EO4)**\| Realised as well I never did a final weigh in before c section! **(3. EO4)**\| Never managed to start up yoga or other exercise beyond daily life needs **(4. EO4)**\| but\| as soon as I am fit and able I must start some activities again.\| Will book in for a video chat in January.\| Thank you for not giving up on me!\| Newborn life is just as I remember it\| but\| I seem to be more used to sleep deprivation **(5. EO5e)**\| so\| I will need to look at some of those mindfulness techniques you sent.\| Breastfeeding mega demanding and sore **(6. EO6)**\| but\| I know will be best in the long run\| (just need to remember to put the vit d drops on before we start (emojis)). **(7. EO4)**\| Whole house is very excited for Christmas,\| (name) is especially keen\| but\| I love it because it keeps me going.\| Hope you're well\| and\| looking forward to Christmas with the family (participant name) x |
| **Message 9** | Hi (coach name),\| I’m doing ok\| thanks\| just really bad on keeping you updated on here **(1. EO3)**. \| I’m really sorry. **(2. EO1)**\| I was wondering going forwards if we could do quick 5-10 mins call update.\| Is this something you can do?\| I’m just very bad at assigning time to sit down and write (3.1)\| as\| always get distracted (3.2)\| and\| put it off to later. (3.3) **(3. EO3)**\| No worries if this is not possible.\| The PGP has actually calmed down (4.1)\| which is great (4.2)\| and\| have been able to go out for walks again (4.3)\| and\| generally just move about a bit more (4.4) **(4. EO5i)**\| The midwife has referred me for some physio\| so\| waiting for an appointment to come through.\| I’m also told they might give me belt to wear\| which will help.\| I have noticed this week (5.1)\| (probably because of the heat) (5.2)\| that my fingers / hands & feet are quite swollen. (5.1) **(5. EO2)**\| My water intake has dropped **(6. EO7)**\| so have put a reminder in my work calendar to alert me. **(7. EO5i)**\| Food wise, my portion size is still on the large size. **(8. EO4)**\| Don’t know how to manage this. **(9. EO2)**\| Hope to speak to you soon\| Thanks (participant name) |
| **Message 10** | Hi (coach name),\| Thanks for your advice about proteins,\| I’ve added it to my goals. **(1. EO5i)**\| Unfortunately, I’ve been extremely unwell the last 2 weeks **(2. EO1)**\| which included me collapsing (3.1)\| and\| having a lovely trip to hospital in an ambulance. (3.2) **(3. EO7)**\| My body is fighting a viral infection and shut down. **(4. EO7)**\| I hit my head (5.1)\| and\| have a lovely black eye. (5.2) **(5. EO7)**\| Due to this my goals and everything have taken a backseat. **(6. EO2)**\| I’ve been advised to sit down as much as possible with my feet up.\| I’m struggling with controlling my body temp (7.1)\| so\| am constantly hot (7.2)\| which is making things worse! (7.3) **(7. EO6)**\| I went back to work Thursday (8.1)\| and\| managed ok. (8.2) **(8. EO5i)**\| The baby is causing me to have my first cravings- wholegrain cheerios! **(9. EO7)**\| I’ve just booked in my c section in 5 weeks! (10.1)\| Any advice on how to prepare would be great. (10.2) **(10. EO2)**\| I’ve been signposted to Bluebell to support with my mental health **(11. EO2)**\| although feeling ok at the moment.\| I am starting maternity leave in 2 weeks\| so\| will have a lot more time for me (for a few weeks lol!)\| Sorry for the long message! **(12. EO4)** (participant name) |

# APPENDIX 3: Survey messages’ EOs and subEOs distribution

| **EO categories** | **Patient messages, n (%)** | **Including subEOs, n (%)** |
| --- | --- | --- |
| **EXPLICIT EOS** | *19 (21.8)* | *27 (22.3)* |
| **Positive** | *2 (10.5)* | 2 (7.4) |
| **EO5e – Self-judgement** | 2 (2.2) | 2 (1.6) |
| **Negative** | *17 (89.5)* | 25 (92.6) |
| **EO1 – Feelings** | **7 (7.5)** | **9 (7.1)** |
| **EO3 – Judgement (others or self)** | 4 (4.3) | 7 (5.5) |
| **EO6 – Appreciation (things, events, actions)** | 6 (6.5) | **9 (7.1)** |
| **IMPLICIT EOs** | *68 (78.2)* | *94 (77.7)* |
| **Positive** | *20 (29.4)* | 29 (16.3) |
| **EO5i – Self-judgement** | **20 (21.5)** | **29 (22.8)** |
| **Negative** | *48 (70.6)* | 65 (83.7) |
| **EO2 – Feelings** | 14 (15.1) | 20 (15.7) |
| **EO4 – Judgement (others or self)** | 13 (14.0) | 15 (11.8) |
| **EO7 – Appreciation (things, events, actions)** | 21 (22.6) | **30 (23.6)** |
| **TOTAL** | **87 (100)** | **121 (100)** |
